# Low-Dose Ammonium Preconditioning Enhances Endurance in Submaximal Physical Exercises

**DOI:** 10.3390/sports9020029

**Published:** 2021-02-16

**Authors:** Igor Mindukshev, Julia Sudnitsyna, Nikolay V. Goncharov, Elisaveta Skverchinskaya, Irina Dobrylko, Elena Nikitina, Alexandr I. Krivchenko, Stepan Gambaryan

**Affiliations:** 1 Sechenov Institute of Evolutionary Physiology and Biochemistry, Russian Academy of Sciences, Thorez pr., 44, 194223 Saint-Petersburg, Russia; sudnitsyna@iephb.ru or ngoncharov@gmail.com (N.V.G.); lisarafail@mail.ru (E.S.); dobrilko@mail.ru (I.D.); elena.nikitina@bk.ru (E.N.); allkriv@yandex.ru (A.I.K.); s.gambaryan@klin-biochem.uni-wuerzburg.de (S.G.); 2 Center for Theoretical Problems of Physicochemical Pharmacology, Russian Academy of Sciences, Kosygina st., 4, 119991 Moscow, Russia

**Keywords:** preconditioning, ammonium, cardiopulmonary endurance test, ischemia preconditioning, physical exercise

## Abstract

Preconditioning is often used in medicine to protect organs from ischemic damage and in athletes to enhance the performances. We tested whether low-dose ammonium preconditioning (AMP) could have a beneficial effect on physical exercises (PE). We used Cardiopulmonary Exercise Testing (CPET) on a treadmill to investigate the effects of low-dose AMP on the physical exercise capacity of professional track and field athletes and tested twenty-five athletes. Because of the individual differences between athletes, we performed a preliminary treadmill test (Pre-test) and, according to the results, the athletes were randomly allocated into the AMP and control (placebo, PL) group based on the similarity of the total distance covered on a treadmill. In the AMP group, the covered distance increased (11.3 ± 3.6%, *p* < 0.02) compared to Pre-test. Similarly, AMP significantly increased O_2_ uptake volume—VO_2_ (4.6 ± 2.3%, *p* < 0.03) and pulmonary CO_2_ output—VCO_2_ (8.7 ± 2.8%, *p* < 0.01). Further, the basic blood parameters (pH, pO_2_, and lactate) shift was lower despite the greater physical exercise progress in the AMP group compared to Pre-test, whereas in the placebo group there were no differences between Pre-test and Load-test. Importantly, the AMP significantly increased red blood cell count (6.8 ± 2.0%, *p* < 0.01) and hemoglobin concentration (5.3 ± 1.9%, *p* < 0.01), which might explain the beneficial effects in physical exercise progress. For the first time, we showed that low-dose AMP had clear beneficial effects on submaximal PE.

## 1. Introduction

Training and recovery are fundamental to improve performance in athletes or people working under strenuous exercises (firefighters, industrial abseilers, military men, etc.), thus optimization of training strategies for performance enhancement and the search of the new methods as preconditioning for the endurance enhancement is an important area of research in sports medicine and physical exercise (PE) physiology [1,2,3,4].

Among other techniques, ischemic preconditioning (IPC) has been used extensively in health and disease [5,6,7]. IPC was shown to improve metabolic efficiency by lowering glycogen [8], adenosine triphosphate (ATP) depletion [9], and lactate concentration [10] therefore, attempts were made to use IPC in sports medicine. However, the analysis of the results presented in the recent reviews [11,12] showed that the beneficial effects of IPC were described only in ten out of twenty-two reviewed reports [5,13,14], whereas in twelve publications no effects of IPC on exercise performance were found [11]. Future work is required to find out in which types of exercises IPC might have beneficial effects.

Another method of preconditioning is connected with the prevention of shifts in the acid-base balance because its changes affect blood buffering capacity leading to metabolic acidosis or alkalosis which may affect endurance in extensive and sub-maximal PE [15]. However, preconditioning with lactate [16], taurine [17,18], sodium citrate, sodium bicarbonate or/and ammonium chloride [19,20,21,22,23] had very low, or no direct effects on PE progress [24]. The administration of high levels of ammonium chloride (300 mg/kg) had an even small negative effect on endurance [25]. In response to PE metabolic modifications that affect the equilibrium of the biochemical internal environment occur in the musculoskeletal, cardiovascular, respiratory, endocrine, and immune systems [26,27,28]. During PE, intramuscular accumulation of lactic acid leading to lactic acidosis has long been considered as one of the most important factors in fatigue [29,30]. At the same time, in PE, ammonia/ammonium (Am) is produced and released from the skeletal muscles, and its production increases with intensity and duration of exercise leading to hyperammonemia and consequent ROS formation which makes Am another important factor of exercise-induced contractile dysfunctions, muscle damage, fatigue and complications in rehabilitation [31,32,33,34,35]. To reduce the Am level, amino acids, like glutamate, or α-keto acids supplementation was used and showed beneficial effects and improved training tolerance [36,37,38]. However, preconditioning with a low dose Am supplementation directly before PE had not been considered previously. By analogy with IPC which mobilizes protective systems of the organism and, in contrast to previous studies [25,39] where relatively high dose Am preconditioning (AMP) 300 mg/kg was used, we tested whether the low dose AMP (10 mg/kg) administrated 5 min before the trial could have a beneficial effect on PE.

The mechanisms of adaptation to excessive Am production in exercise are not yet clear. We suggest that professional sportsmen have enhanced adaptation mechanisms likewise for hypoxia for Am homeostasis in blood and Am detoxification systems. We hypothesize that activation of such systems directly prior the exercise could enhance the working efficiency of sportsmen and avoid/soften the metabolic stress as it was shown for rats [40,41,42]. Here, we tested this hypothesis by adopting the new approach of ammonium preconditioning by the administration of low-dose exogenous ammonium chloride. We used cardiopulmonary endurance testing (CPET) of professional athletes undergoing exercise, with or without AMP, and showed that AMP extended the covered distance (D) compared to the placebo-administered control group (PL).

## 2. Materials and Methods

### 2.1. Ethics Approval

All procedures and experimental protocols were submitted and approved by the Ethical Committee of Sechenov Institute of Evolutionary Physiology and Biochemistry of the Russian Academy of Sciences (protocol no. 7 from 10.02.14) and conducted in accordance with the Declaration of Helsinki. All participants were fully familiarized with the laboratory exercise procedures before the data collection. All participants were fully instructed about the testing procedures and gave their written informed consent before inclusion.

### 2.2. Participants

Twenty-five professional male athletes participated in this study. All selected sportsmen were track and field athletes (sprint, long-distance run, hurdling, high jumps, all-round, triathlon, marathon run), after summer encampment, in the autumn rhythm of their training cycle. All subjects got a full medical checkup twice a year. None of them were smokers, used medication at least two weeks before the trials, and demonstrated any pathological findings during the pre-medical examination. During the trials, the participants were instructed not to drink caffeine, maintain their typical hydration and food ingestion habits.

### 2.3. Experimental Design

**Figure 1:**
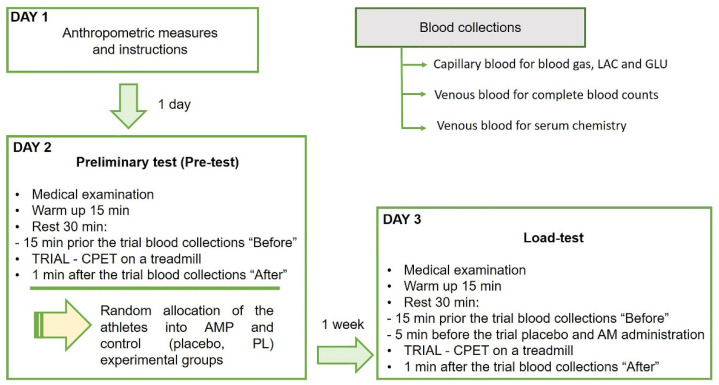
The design of the randomized controlled trial of low dose ammonium preconditioning (AMP) in submaximal exercise. The trial was designed as a single-blind pre-post study with experimental and control groups, and a randomized allocation of 25 well-trained participating athletes. The same exercise protocol on a treadmill with CPET was used for the preliminary (Pre-test) and main preconditioning test (Load-test) to compare performance and biomarkers responses. The group allocation was based on the covered in Pre-test on a treadmill distance (D) as the marker of endurance. Blood was drawn in three tubes for blood gas, lactate (LAC) and glucose (GLU), for complete blood counts and for serum chemistry, before and after each trial.

In this study, we performed a single-blind randomized controlled pre-post trial with experimental and control groups using the same physical exercise protocol to compare performance and biomarkers responses (Figure 1). During the study, the participants visited the laboratory three times, one for the anthropometric measures and experiment familiarization, another in one day for the preliminary test, and the last one in one week for the single-blind load-test. The anthropometric measures as age, weight, and height, and experience of each professional athlete are presented in Table 1.

#### 2.3.1. Preliminary Test (Pre-Test)

**Figure 2:**
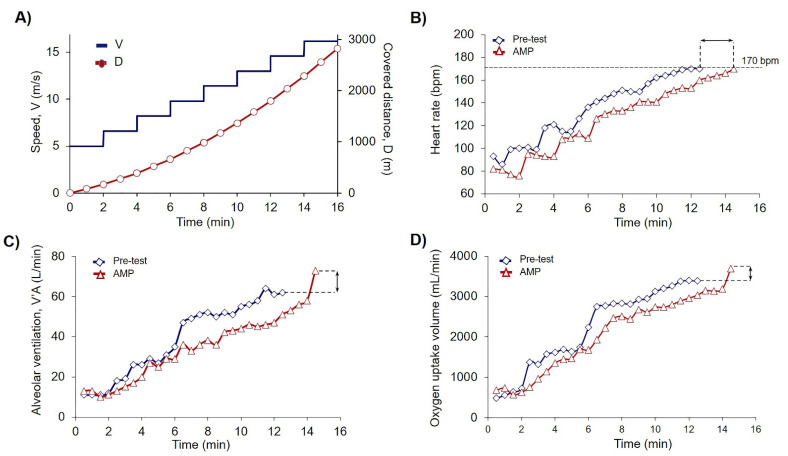
Low-dose AMP increased PE capacity. (**A**) Scheme of PE test where V is speed dynamics (m/s), and D is calculated covered distance (m). According to the experimental design (2.3) exercises were terminated when the submaximal heart rate (HR) achieved the cessation criteria of 170 beats per minute (bpm); (**B–D**) Original records of single athlete of AMP group achieved the cessation criteria of 170 bpm during the trial on the treadmill in Pre-test (blue squares) and Load-test-AMP (red triangles); (**B**) Dynamics of the heart rate (HR); (**C**) Dynamics of alveolar ventilation (V’A); and (**D**) Dynamics of O_2_ uptake volume (V’O_2_). Black arrows indicate the differences between measured parameters in the Pre-test and AMP for a single athlete of AMP group.

Each trial day started at 9.00 am. Before each trial, all participants were examined by the physiotherapist for their physical condition. Next was an obligatory 15 min warmup, followed by 30 min rest for the recovery. Venous blood was collected from each athlete into three tubes 15 min prior to the trial. The preliminary walking test on a treadmill with cardiopulmonary endurance test (CPET) was performed to allocate the athletes into AMP and control (placebo, PL) groups according to the covered on a treadmill distance (D). Each trial consisted of eight steps, 2 min duration each, with an initial treadmill velocity of 5 km/h and 0% incline. For each subsequent step, the speed was increased by 1.6 km/h. Exercises were terminated when the heart rate (HR) achieved 170 bpm (Figure 2). The recovery took 1 min at a treadmill speed of 5 km/h. After the trial, another three tubes of blood were drawn.

#### 2.3.2. Cessation Criteria for the Trial

For the trial termination, the following safety precautions were set up: (1) HR attainment of 170 bpm. The level of 170 bpm was calculated from 85% of peak heart rate for an average age of 20 years [43,44] in order not to stress the participants and not to disturb their training cycle, (2) weakness, dizziness, (3) athlete’s denial, (4) HR disturbance, (5) ST-segment displacement in the cardiogram. According to these criteria, no one of the tested athletes was terminated during the exercise.

#### 2.3.3. Methodology of Group Formation

After the test for individual functional parameters (Pre-test) the athletes were randomly selected into two groups, AM preconditioning and placebo (Table 1), according to the similarity of the covered on the treadmill distance (D) before reaching the maximal set for this study heart rate of 170 bmp. The AMP group consisted of 14 athletes (mean D 1871 ± 206 m) and the PL group consisted of 11 athletes (mean D 1873 ± 244 m).

#### 2.3.4. Testing Day

Participants returned to the laboratory in one week to complete the blind trial with preconditioning. As previously described, before each trial, all participants were examined by the physiotherapist for their physical condition. Next was an obligatory 15 min warmup, followed by 30 min rest for the recovery. Venous blood was collected from each athlete into three tubes 15 min prior to the trial. Then, 5 min before the trial all athletes were given according to their group 10 mL of preconditioning medication following by washing down with sparkling water. Each trial consisted of eight steps, 2 min duration each, with an initial treadmill velocity of 5 km/h and 0% incline. For each subsequent step, the speed was increased by 1.6 km/h. Exercises were terminated when the heart rate (HR) achieved 170 bpm (Figure 2). The recovery took 1 min at a treadmill speed of 5 km/h. After the trial, another three tubes of blood were drawn.

#### 2.3.5. Ammonium and Placebo Preconditioning

Five minutes prior to the trial each athlete of the AMP group was orally administered 10 mg/kg ammonium chloride NH_4_Cl (Sigma-Aldrich, Munich, Germany) dissolved in 10 mL water, following by washing down with sparkling water. To assure the blind experiment, CaCl_2_ (Sigma-Aldrich, Munich, Germany) dissolved in 10 mL water, 5 mg/kg, was administered to the control placebo group, following by washing down with sparkling water. CaCl_2_ was chosen for the control placebo group based on the similarity of bitter taste with NH_4_Cl.

### 2.4. Instrumentation and Measurements

#### 2.4.1. Cardiopulmonary Endurance Test (CPET)

During the cardiopulmonary exercise testing, all respiratory data were collected with an ergospirometer OxyconPro (Vyaire (Jaeger), Höchberg, Germany) calibrated daily in the morning before the testing (according to the manufacturer instruction). The tests were performed on a treadmill T2100 (GE Medical Systems Information Technologies GmbH, Freiburg im Breisgau, Germany) in a calm illuminated room (17–22 °C, ambient conditions) in accordance to standardized analysis recommended by the manufacturer. Before testing each athlete spent 30 min in a sitting position being instructed on experimental design. Bisternal electrocardiographic electrodes were mounted in 12 derivations. An arm blood pressure cuff was fixed, as well as a face-piece with a flow sensor connected to a gas analysis indicator and a volume sensor. During the test, the 12-lead electrocardiogram (ECG) and heart rate (HR) were monitored continually, while the arterial blood pressure (ABP) was measured at 2 min intervals. Analysis of the respiratory cycle in the “breath-by-breath” mode, based on a 10 s interval, registered the following parameters: mixed expiratory CO_2_ pressure (PECO_2_), end-expiratory CO_2_ pressure (PET-CO_2_), O_2_ uptake volume (VO_2_), pulmonary CO_2_ output (VCO_2_), alveolar ventilation (V’A), breathing reserve (BR), and respiratory exchange ratio (RER).

#### 2.4.2. Blood Collections and Analyses

Venous blood was collected from the median cubital vein 15 min before Pre-test and Load-test and immediately after them, four times for each athlete in total. The blood was drawn into three tubes. 175 µL Blood gas capillaries from PET (Sarstedt AG & Co., Nümbrecht, Germany) were used for lactate (LAC) and glucose (GLU) concentration and acid-base balance (ABB), including base excess (BEb) analyses by Cobas b 221 system modification 6 (Roche Diagnostics GmbH, Mannheim, Germany). 1 mL VACUETTE^®^ tubes K2E K2EDTA (Greiner Bio-One GmbH, Frickenhausen, Germany) were used for complete blood counts (CBC): red blood cell count, RBC; mean corpuscular volume of red blood cells, MCV; hemoglobin (Hb) concentration, HGB; hematocrit, HCT; platelet count, PLT; white blood cells count, WBC; lymphocyte count, LYM, by Medonic-M20 (Boule Medical A.B., Stockholm, Sweden). 5 mL VACUETTE^®^ CAT Serum Separator Clot Activator tubes (Greiner Bio-One GmbH, Frickenhausen, Germany) were used for serum chemistry analyses: glucose concentration (GLU), the activity of alanine aminotransferase (ALT), aspartate aminotransferase (AST), gamma-glutamyltransferase (GGTP), lactate dehydrogenase (LDH), by Architect c8000 analyzer (Abbott, Abbott Park, IL, USA). All blood analyses were performed in a clinical diagnostic laboratory of Saint Petersburg Almazov National Medical Research Centre (blood gas, LAC, and GLU) and in a clinical diagnostic laboratory of Saint Petersburg Clinical Hospital of Russian Academy of Sciences (complete blood counts and serum biochemistry) on analyzers that had undergone quality control to ensure high-quality results. All devices were certificated for blood laboratory analyses.

### 2.5. Data Calculations

The increment of the parameter Δ(A)% was estimated from the following equation:

Δ(A)% = 100 × (A_Load-test_ − A_Pre-test_)/A_Pre-test_ (1)

where A_Pre-test_—The parameter of the preliminary test, A_Load-test_—The parameter of PE test (AMP or PL).

The differences between the parameters before and after the PE were calculated for each athlete using the following equation:

ΔX = X_after_ − X_before_ (2)

where X_after_—The parameter after the PE, X_before_—The parameter before the PE.

### 2.6. Statistical Analysis

Descriptive statistics for participant characters and dependent variables are shown as means ± standard deviation (M ± SD). The data were normally distributed as assessed by Shapiro-Wilk’s normality test (*p* > 0.05), except enzymatic parameters which were log-transformed. For functional parameters (Table 2), the 2 × 2 mixed analysis of variance (ANOVA) for groups (AMP, PL) by time (Pre-test, Load-test) was used. For the blood parameters (Table 3, Table 4, Table 5 and Table 6), the 2 × 2 mixed analysis of variance (ANOVA) for groups (AMP, PL) by time (“before”, “after”) was used to examine interactions of the independent variables (as AMP or PL) on the dependent variables (as Hb). The assumption of sphericity was confirmed for the two-way interaction by Mauchly’s test of sphericity. The paired *t*-test was used to determine significant differences in parameters “before” and “after” within the group. Statistical analysis was computed using IBM SPSS Statistics v. 23 (IBM Corp., Armonk, N.Y., USA), with an established alpha level of 0.05.

## 3. Results

### 3.1. Ammonium Preconditioning Showed the Beneficial Effects in Physical Exercise Progress

To show the effects of AMP, we compared each athlete’s results of CPET for Pre-test and Load-test. For the explanation of the data in Table 2 here we presented the data of pre-post trial results for one athlete from the AMP group (Figure 2B–D). The quantification of all sportsmen CPET data including covered distance is summarized in Table 2. Analysis of covered distance data for each participant is presented in Supplementary Materials Table S1. AMP significantly increased D (*p* < 0.002), VO2 (*p* < 0.05), and VCO2 (*p* < 0.03), whereas the differences in PL group were not significant (Table 2).

**Figure 3:**
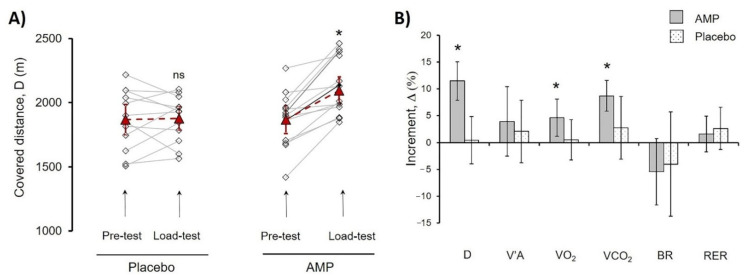
Comparison of AMP and PL group parameters in treadmill PE. (**A**) Progress of distance (D) covered by each athlete in Pre-test and Load-test for PL and AMP groups. Lines indicate the progress for each athlete; dash lines indicate mean values. (**B**) Mean values of the following parameters increment (%) for AMP and PL groups, alveolar ventilation (V’A), O_2_ uptake volume (VO_2_), pulmonary CO_2_ output (VCO_2_), breathing reserve (BR), respiratory exchange ratio (RER). Data in (A) are presented as Mean ± SD, * *p* < 0.05 (Load-test compared to Pre-test). Data in (B) were calculated according to Equation (1) and are presented as Mean ± SD, * *p* < 0.05 (Load-test compared to Pre-test); ns—differences are not significant.

Next, we calculated the D of the Pre-test and Load-test for each athlete (Figure 3A. For evaluation of the differences between AMP and PL groups, the increment was calculated (Equation (1)) and designated as delta (Δ%), where Pre-test values were taken as 100% (Figure 3B).

The AMP significantly increased Δ(D)% by 11.5 ± 3.6% (*p* < 0.01), whereas there were no significant changes in the PL group indicating that CaCl_2_ administration had no effect. Additionally, values of Δ% VO_2_ (4.6 ± 3.3%, *p* < 0.03), and Δ% VCO_2_ (8.7 ± 2.8%, *p* < 0.01) showed significant increase in AMP compared to PL group (Figure 3). Presented results pointed out the clear beneficial effect of low-dose AMP.

### 3.2. AMP Reduced the Shifts in the Acid-Base Balance under Physical Exercises

During Pre-test and Load-test we detected significant blood acidification (pH), increased pCO_2_, and lactate level (LAC), and decreased pO_2_, whereas there were no significant changes in BEb and glucose (GLU) levels (Table 3). It should be mentioned that basal lactate level was slightly increased due to 15 min warmup procedure that was obligatory 30 min prior to each trial.

To calculate the differences between AMP and PL groups we used 2-way ANOVA and detected that the shifts in pH (F1.23 = 4.71; ɳ2 = 0.17; *p* = 0.04), pO_2_ (F1.23 = 4.40; ɳ2 = 0.16; *p* = 0.047), and LAC (F1.23 = 4.75; ɳ2 = 0.17; *p* = 0.040) were significantly lower in case of AMP (Table 3 and Table 4). Thus, even though D in the AMP group was higher (Table 2), AMP reduced the shift in the basic acid-base balance parameters (pH, pO_2_, LAC) compared to the PL group.

### 3.3. AMP Did Not Induce Significant Changes in Main Enzymatic Biochemical Blood Parameters

The activity of ALT, AST, GGTP, and LDH is usually monitored during PE, therefore we evaluated whether AMP affected the activity of these enzymes. Pre-test and Load-test significantly increased the activity of ALT and AST in both, AMP, and PL groups. However, AMP did not cause any changes in enzyme activity (Table 5).

### 3.4. AMP Increased RBC Count and Hb Concentration

PE might cause significant changes in different blood cells concentrations [45], therefore we tested whether AMP could affect these parameters. PE significantly increased WBC and LYM in both, AMP, and PL groups without any changes in MCV and PLT. RBC and HGB were not changed in Pre-test, however these parameters (RBC: F1.23 = 5.03, ɳ2 = 0.18, *p* = 0.035; HGB: F 1.23 = 4.94, ɳ2 = 0.22, *p* = 0.036) significantly increased in AMP compared to PL group (Table 6). At the same time WBC (F1.23 = 0.02; ɳ2 = 0.00; *p* = 0.89) and LYM (F1.23 = 0.04; ɳ2 = 0.00; *p* = 0.95) were not affected by AMP. Using the Equation (2) we calculated the increment (Δ%) of RBC and HGB increase after AMP taking concentration “before” Load-test as 100%. AMP increased RBC and HGB by 6.8 ± 2.0% and 5.3 ± 1.9% (*p* < 0.01 for both) respectively, whereas in PL group there were no changes (Table 4).

## 4. Discussion

Exercises increase the blood ammonia/ammonium level, and the rate of Am accumulation in the blood directly depends on exercise intensity and duration [32,42,46,47]. At rest, the Am level in plasma is maintained from 10 to 60 µM [48,49,50], while during exercises the Am level increases several-fold up to 250 µM, and such state is referred to as exercise-induced hyperammonemia [51,52]. The excess Am level in the blood has clear negative effects [31,32,51]. The exercise produced excess of Am alters neuromuscular activity, contributes to muscle fatigue [31], and increases reactive oxygen species (ROS) generation in mitochondria [53]. The value of Am level in the blood was found to directly correlate with an athlete’s exercise capacity [54]. Therefore, excessive Am production in muscle induces oxidative stress, similar to that in hypoxic states and ischemia/reperfusion, and leads to contractile dysfunctions, muscle weakness, fatigue, and possibly contributes to the complications in recovery [32,33].

The most important finding of our experiments is the increase in RBC count and Hb concentration in the AMP compared to the PL group (Table 4 and Table 6). It is well-known that Am concentration in RBCs exceeds severalfold the concentration in plasma [49,50]. During long-term storage of blood in blood banks, the Am concentration increased in plasma up to 0.5 mM [55] and it was shown that RBCs could transport Am inside the cells [56,57,58,59,60] lowering its concentration in extracellular media [50], indicating that RBCs can not only transport but also trap Am, thus playing a significant role in maintaining Am homeostasis. Physical exercises were reported to cause efflux of RBCs from the spleen [45,61,62,63]. In our experiments, such a fast increase of RBC concentration in the AMP group was most likely also caused by erythrocyte release from the spleen. Accordingly, we speculated that a significant increase of D, VO_2_, and VCO_2_ (Table 2, Figure 3B) is the result of RBC accrual in the AMP group. The capability to sustain high-intensity exercise was shown to depend mostly on the body’s ability to minimize increases in cellular and blood hydrogen ion concentration [39,64]. In our experiments, despite a significant increase in PE progress in the AMP group, the shifts in the acid-base balance (ΔpH, ΔpO_2_, ΔpCO_2,_ and ΔLAC) were lower (Table 3 and Table 4). Therefore, our results indicated the beneficial effects of AMP on maintaining the acid-base balance thus promoting endurance during exercise. As we [50,60,65] and others [56,57,58,59] showed previously, human RBCs can import AM thus possibly lowering the metabolic stress and preventing Am-induced damage of muscle cells by ROS. Therefore, we assume RBCs to play an important role in AM homeostasis and maintenance of the acid-base balance during the PE, however, this idea merits future examinations.

## 5. Conclusions

We still know very little about the adaptation to excessive Am production in exercise, and the mechanisms of such adaptation are not yet clear. We assume that professional sportsmen have enhanced adaptation mechanisms, as for hypoxia [5,11,15,66,67], for Am homeostasis maintenance in blood and Am detoxification systems. We hypothesize that activation of adaptive systems prior to commencement of exercise could enhance the working capacity of sportsmen by softening the metabolic stress consequences. Preconditioning is widely used in different applications [11,14,68,69] and previously the high doses Am preconditioning was tested without any beneficial effects [25,39]. For the first time, here we showed that low-dose AMP had clear beneficial effects on submaximal PE. Our results could not predict yet in which particular type of PE AMP may have beneficial effects, however, we assume that the low-dose AMP could be used for athletes instead of, or together with IPC or remote IPC, in work-out sessions. Thus, such preconditioning could have beneficial effects in people under physical stress (military men, alpinists, firefighters, emergency response workers, etc.). We are confident that our new finding is very important for a better understanding of Am metabolism and Am detoxification system functioning in processes connected to physical activity.

## Figures and Tables

**Table 1:** The anthropometric measures of each athlete, preliminary test results (Pre-test), and formation of ammonium preconditioning (AMP) and control (placebo, PL) groups according to the Pre-test results.

Athletes	Age (years)	Weight (kg)	Height (cm)	Experience (years)	Pre-Test (D, m)	Assigned Group for the Load-Test	
						AMP (D, m)	PL (D, m)
No. 1	18	80	178	8	1417	1417	
No. 2	19	62	174	4	1508		1508
No. 3	18	68	180	8	1522		1522
No. 4	19	66	177	7	1623		1623
No. 5	21	60	171	12	1674	1674	
No. 6	19	61	174	6	1684	1684	
No. 7	28	83	174	12	1706	1706	
No. 8	19	72	179	8	1746		1746
No. 9	20	72	176	7	1853		1853
No. 10	18	72	173	8	1861	1861	
No. 11	18	59	178	6	1861	1861	
No. 12	25	75	186	12	1889	1889	
No. 13	19	70	173	11	1901	1901	
No. 14	18	78	182	6	1901		1901
No. 15	19	75	174	11	1922	1922	
No. 16	19	83	178	10	1950	1950	
No. 17	20	68	178	9	1962	1962	
No. 18	19	67	178	9	1995		1995
No. 19	24	74	178	11	2023	2023	
No. 20	28	75	178	15	2039		2039
No. 21	20	72	174	8	2080	2080	
No. 22	27	82	181	6	2096		2096
No. 23	18	78	182	6	2096		2096
No. 24	18	69	181	6	2218		2218
No. 25	20	72	174	5	2270	2270	
n	25	25	25	25	25	14	11
Mean	20.4	71.7	177.2	8.4	1871.9	1871 ^ns^	1873 ^ns^
SD	3.2	6.9	3.6	2.7	218.8	206	244

Note. D—Covered distance; AMP—Ammonium preconditioning group, PL—Placebo group, n—number of participants, SD—standard deviation. Data were analyzed by independent samples *t*-test, *p* < 0.05, ns, not significant.

**Table 2:** AMP significantly extended D, VO_2_, VCO_2_ among the CPET (cardiopulmonary endurance testing) parameters at the cessation point of 170 bpm. Data are presented as Mean ± SD.

CPET Parameter	Pre-Test		Load-Test	
	AMP	PL	AMP	PL
D, m	1871 ± 206	1872 ± 244	2118 ± 216 *	1885 ± 186
V’A, L/min	77.0 ± 14.6	79.0 ± 15.9	83.2 ± 12.6	81.4 ± 13.3
VO_2_, mL/min	3292 ± 482	3398 ± 456	3433 ± 405 *	3410 ± 382
VCO_2_, mL/min	2775 ± 423	2903 ± 481	3084 ± 397 *	2946 ± 432
BR, %	50.4 ± 9.1	47.7 ± 11.5	48.1 ± 8.7	47.4 ± 15.3
RER	0.84 ± 0.04	0.85 ± 0.07	0.90 ± 0.04	0.86 ± 0.06

Abbreviations: AMP—Ammonium preconditioning group, PL—Placebo group, D—Covered distance, V’A—Alveolar ventilation, VO_2_—O_2_ uptake volume, VCO_2_—Pulmonary CO_2_ output, BR—Breathing reserve, RER—respiratory exchange ratio. Data were analyzed by 2-way mixed analysis of variance (ANOVA) for the group (AMP, PL) by time (Pre-test, Load-test), * *p* < 0.05 compared to AMP in Pre-test.

**Table 3:** AMP significantly reduced the shifts in acid-base balance parameters, pH, pO_2,_ and lactate. Data are presented as Mean ± SD.

Parameter	Pre-Test				Load-Test			
	AMP		PL		AMP		PL	
	Before	After	Before	After	Before	After	Before	After
pH	7.37 ± 0.04	7.29 ± 0.07 *	7.39 ± 0.02	7.31 ± 0.05 *	7.35 ± 0.03	7.30 ± 0.04 *	7.36 ± 0.04	7.28 ± 0.05 *
pO_2_ (mmHg)	39.6 ± 8.0	34.6 ± 6.4 *	38.5 ± 6.7	31.7 ± 8.5 *	38.4 ± 3.8	34.0 ± 7.0 *	40.3 ± 3.2	29.9 ± 7.9 *
pCO_2_ (mmHg)	49.8 ± 8.4	58.3 ± 10.2 *	46.5 ± 6.3	57.6 ± 8.8 *	50.1 ± 6.6	55.7 ± 9.8 *	51.5 ± 6.7	60.4 ± 9.7 *
BEb (mM)	1.81 ± 1.95	0.57 ± 2.08 *	2.51 ± 3.25	1.14 ± 2.99	1.74 ± 3.27	−0.42 ± 2.27	2.26 ± 2.81	0.82 ± 2.54
GLU (mM)	5.15 ± 0.43	5.08 ± 0.86	4.95 ± 0.79	5.29 ± 0.84	4.77 ± 0.69	5.64 ± 0.81 *	4.89 ± 0.81	5.05 ± 0.66
LAC (mM)	3.71 ± 0.76	6.53 ± 0.70 *	3.14 ± 0.79	6.30 ± 0.88 *	3.82 ± 0.62	5.69 ± 1.08 *	3.41 ± 0.44	6.18 ± 0.81 *

Note: pO_2_, the partial pressure of O_2_; pCO_2_, the partial pressure of CO_2_; BEb, base excess; GLU, glucose concentration; LAC, lactate concentration. The data were calculated by 2-way ANOVA for a group (AMP, PL) by time (“before”, “after”). Paired *t*-test was used to evaluate the differences between “before” and “after”, * *p* < 0.05.

**Table 4:** AMP significantly increased RBC and HGB and lowered the shifts in acid-base balance parameters (ΔpH, ΔpO_2_, ΔpCO_2_) and LAC (ΔLAC). Data are presented as M ± SD.

Parameter	Pre-Test		Load-Test	
	AMP n = 14	PL n = 11	AMP n = 14	PL n = 11
Δ RBC × 10^12^ (cells/L)	0.10 ± 0.10 *	0.06 ± 0.11	0.34 ± 0.1 *	0.05 ± 0.05
(%)	(2.0 ± 2.0)	(1.2 ± 2.1)	(6.8 ± 2.0)	(1.0 ± 1.0)
Δ HGB (g/L)	3.8 ± 2.5 *	2.5 ± 1.4	7.8 ± 2.8 *	1.2 ± 1.6
(%)	(2.6 ± 1.6)	(1.7 ± 1.0)	(5.3 ± 1.9)	(0.8 ± 1.1)
ΔpH	−0.08 ± 0.03 *	−0.08 ± 0.03	−0.05 ± 0.02 *	−0.08 ± 0.02
ΔpO_2_ (mmHg)	−6.2 ± 5.1 *	−6.5 ± 5.6	−3.9 ± 2.3 *	−10.7 ± 4.5
ΔpCO_2_ (mmHg)	7.7 ± 3.7 *	10.9 ± 4.3	5.5 ± 3.1 *	10.8 ± 3.2
ΔLAC (mM)	2.8 ± 0.6 *	3.2 ± 0.7	1.9 ± 0.6 *	2.8 ± 0.7

Note. AMP—Ammonium preconditioning group, PL—Placebo group, pO_2_—Partial pressure of O_2_, pCO_2_—Partial pressure of CO_2_, LAC—Lactate, HGB—Hemoglobin concentration, RBC—Red blood cell count; Δ indicates the differences between the parameters before and after the physical exercise (calculated for each athlete using the Equation (2)), % indicates the increment of the parameters (calculated using the Equation (1)). Data were calculated by paired Student’s *t*-test (Pre-test and Load-test for AMP or PL), * *p* < 0.05.

**Table 5:** Physical exercise significantly increased ALT and AST, whereas AMP had no effect on measured enzymatic activities. Data are presented as Mean ± SD.

Parameter	Pre-Test				Load-Test			
	AMP		PL		AMP		PL	
	Before	After	Before	After	Before	After	Before	After
ALT (U/L)	25.9 ± 13.7	29.1 ± 14.2 *	23.3 ± 14.4	27.2 ± 17.9 *	24.8 ± 14.1	30.3 ± 13.3 *	24.6 ± 14.9	28.7 ± 17.5 *
AST (U/L)	23.1 ± 9.6	25.4 ± 9.8 *	20.3 ± 12.3	23.0 ± 12.6 *	26.0 ± 18.9	31.5 ± 21.2 *	21.2 ± 13.4	24.3 ± 12.7 *
GGTP (U/L)	15.9 ± 5.4	16.4 ± 5.5	14.3 ± 7.83	14.9 ± 8.2	17.4 ± 5.0	19.5 ± 5.5	14.8 ± 7.0	16.8 ± 8.9
LDH (U/L)	268 ± 51	277 ± 39	266 ± 51	269 ± 56	220 ± 43	236 ± 18	221 ± 47	229 ± 55

Note: ALT—The activity of alanine aminotransferase; AST—The activity of aspartate aminotransferase; GGTP—The activity of gamma-glutamyltransferase; LDH—The activity of lactate dehydrogenase; The data were calculated by 2-way ANOVA for a group (AMP, PL) by time (“before”, “after”). Paired *t*-test was used to evaluate the differences between “before” and “after”, * *p* < 0.05.

**Table 6:** AMP significantly increased Hb and RBC among the measured blood parameters. Data are presented as Mean ± SD.

Parameter	Pre-Test				Load-Test			
	AMP		PL		AMP		PL	
	Before	After	Before	After	Before	After	Before	After
HGB (g/L)	152.7 ± 3.1	156.5 ± 4.6	143.0 ± 2.0	146.6 ± 2.2	149.5 ± 4.0	157.3 ± 3.3 *	148.0 ± 2.4	148.0 ± 2.3
RBC (cells/L)	5.14 ± 0.12	5.24 ± 0.17	4.91 ± 0.07	4.99 ± 0.11	5.02 ± 0.16	5.35 ± 0.14 *	4.98 ± 0.10	5.05 ± 0.09 *
MCV (fL)	84.5 ± 0.5	84.2 ± 0.5	83.5 ± 0.5	83.3 ± 0.5	84.4 ± 0.4	84.4 ± 0.5	83.7 ± 0.5	83.9 ± 0.6
WBC (cells/L)	6.8 ± 0.6	8.4 ± 0.9 *	6.8 ± 0.4	8.4 ± 0.5 *	7.0 ± 0.6	9.9 ± 0.9 *	7.6 ± 0.9	9.7 ± 1.1 *
LYM (cells/L)	2.1 ± 0.2	2.7 ± 0.3 *	2.2 ± 0.2	3.0 ± 0.4 *	2.0 ± 0.2	3.2 ± 0.3 *	2.1 ± 0.2	3.2 ± 0.4 *
PLT (cells/L)	204 ± 29	242 ± 24	261 ± 12	266 ± 28	186 ± 43	218 ± 41	231 ± 34	217 ± 50

Note: HGB—Hemoglobin concentration; RBC—Red blood cell count; MCV—Mean corpuscular volume of erythrocytes; WBC—White blood cells count; LYM—Lymphocytes count; PLT—Platelet count. The data were calculated by 2-way ANOVA for a group (AMP, PL) by time (“before”, “after”). Paired *t*-test was used to evaluate the differences between “before” and “after”, * *p* < 0.05.

## Data Availability

The data presented in this study are available on request from the corresponding author. The data are not publicly available due to privacy reasons.

## Supplementary Materials

The following are available online at https://www.mdpi.com/2075-4663/9/2/29/s1, Supplementary Table S1: AMP significantly increased covered distance D.
