# Supplementary material for: Low-Dose Ammonium Preconditioning Enhances Endurance in Submaximal Physical Exercises
Source: Sports (Basel). 2021 Feb 16;9(2):29. doi: 10.3390/sports9020029 (PMC7920466; doi:10.3390/sports9020029)
Supplement: Supplementary file 1 [file sports-09-00029-s001.pdf]

sports

MDPI

Supplementary Material

Low-Dose Ammonium Preconditioning Enhances Endurance in Submaximal Physical Exercises

Igor Mindukshev [1,*,†], Julia Sudnitsyna [1,2,†], Nikolay V. Goncharov [1], Elisaveta Skverchinskaya [1], Irina Dobrylko [1], Elena Nikitina [1], Alexandr I. Krivchenko [1] and Stepan Gambaryan [1]

[1] Sechenov Institute of Evolutionary Physiology and Biochemistry, Russian Academy of Sciences, Thorez pr., 44, 194223 Saint-Petersburg, Russia; sudnitsyna@iephb.ru or julia.sudnitsyna@gmail.com (J.S.); ngoncharov@gmail.com (N.V.G.); lisarafail@mail.ru (E.S.); dobrilko@mail.ru (I.D.); elena.nikitina@bk.ru (E.N.); allkriv@yandex.ru (A.I.K.); s.gambaryan@klin-biochem.uni-wuerzburg.de (S.G.)

[2] Center for Theoretical Problems of Physicochemical Pharmacology, Russian Academy of Sciences, Kosygina st., 4, 119991 Moscow, Russia

* Correspondence: iv_mindukshev@mail.ru; Tel.: +7-921-979-4793

† These authors contributed equally to this work.

Citation: Mindukshev, I.; Sudnitsyna, J.; Goncharov, N.V.; Skverchinskaya, E.; Dobrylko, I.; Nikitina, E.; Krivchenko, A.I.; Gambaryan, S. Low-Dose Ammonium Preconditioning Enhances Endurance in Submaximal Physical Exercises. *Sports* **2021**, *9*, 29. https://doi.org/10.3390/sports9020029

Academic Editor: Dale W. Chapman

Received: 30 December 2020
Accepted: 10 February 2021
Published: 16 February 2021

Publisher's Note: MDPI stays neutral with regard to jurisdictional claims in published maps and institutional affiliations.

Copyright: © 2021 by the authors. Licensee MDPI, Basel, Switzerland. This article is an open access article distributed under the terms and conditions of the Creative Commons Attribution (CC BY) license (http://creativecommons.org/licenses/by/4.0/).

Table S1. AMP significantly increased covered distance D. Data are presented as Mean ± standard deviation (SD).

**Athletes**	**AMP**		**Placebo**	
	Pre-test	Load-test	Pre-test	Load-test
	Covered distance, D (m)			
No. 1	1417	1849		
No. 2			1508	1703
No. 3			1522	1565
No. 4			1623	1845
No. 5	1674	2011		
No. 6	1684	1885		
No. 7	1706	1877		
No. 8			1746	1966
No. 9			1853	1601
No. 10	1861	1991		
No. 11	1861	2137		
No. 12	1889	1966		
No. 13	1901	2169		
No. 14			1901	1930
No. 15	1922	2399		
No. 16	1950	1987		
No. 17	1962	2417		
No. 18			1995	2104
No. 19	2023	2462		
No. 20			2039	1962
No. 21	2080	2129		
No. 22			2096	2080
No. 23			2096	1934
No. 24			2218	2047
No. 25	2270	2368		
n	14	14	11	14
Mean	1871	2118 **#	1873 ^{ns}	1885 ^{ns}#
SD	206	216	244	186

Abbreviations: AMP—ammonium preconditioning group, PL—placebo group, D—covered distance, n—number of participants, SD—standard deviation. Data were analyzed by paired t-test, **, $p < 0.0001$, ns—not significant, $p > 0.05$; and by independent samples t-test, #, $p < 0.008$.
